# Wolcott-Rallison Syndrome, a Rare Cause of Permanent Diabetes Mellitus in Infants—Case Report

**DOI:** 10.3390/pediatric15040056

**Published:** 2023-10-16

**Authors:** Alexandru-Ștefan Niculae, Claudia Bolba, Alina Grama, Alexandra Mariş, Laura Bodea, Simona Căinap, Alexandra Mititelu, Otilia Fufezan, Tudor Lucian Pop

**Affiliations:** 1 2nd Pediatric Discipline, Department of Mother and Child, Iuliu Hatieganu University of Medicine and Pharmacy Cluj-Napoca, 400177 Cluj-Napoca, Romania; niculae.alexandru.stefan@elearn.umfcluj.ro (A.-Ș.N.); simona.cainap@gmail.com (S.C.); perta_alexandra@yahoo.com (A.M.); tudor.pop@umfcluj.ro (T.L.P.); 2 2nd Pediatric Clinic, Emergency Clinical Hospital for Children Cluj-Napoca, 400177 Cluj-Napoca, Romania; claudine.bolba@yahoo.com; 3 Intesive Care Unit, Emergency Clinical Hospital for Children Cluj-Napoca, 400370 Cluj-Napoca, Romania; alexandramaris85@gmail.com (A.M.); bodea.laura@yahoo.com (L.B.); 4 Department of Imaging, Emergency Clinical Hospital for Children Cluj-Napoca, 400370 Cluj-Napoca, Romania; otilia.fufezan@gmail.com

**Keywords:** Wolcott-Rallison syndrome, diabetes, permanent neonatal diabetes, insulin therapy, long-acting insulin analogs

## Abstract

Wolcott-Rallison syndrome is a rare cause of permanent neonatal diabetes mellitus caused by mutations in the eukaryotic translation initiation factor 2 alpha kinase 3 gene (EIF2AK3). Individuals affected by this disorder have severe hyperglycemia, pancreatic failure, and bone abnormalities and are prone to severe and life-threatening episodes of liver failure. This report illustrates the case of a 2-month-old infant with extreme hyperglycemia and severe diabetic ketoacidosis. Acute management was focused on correcting severe acidosis. Further management aimed to obtain stable blood glucose levels, balancing the patient’s need for comfort and lack of distress with the clinicians’ need for adequate information regarding the patient’s glycemic control. Genetic testing of the patient and his parents confirmed the diagnosis. The follow-up for 18 months after diagnosis is detailed, illustrating both the therapeutic success of subcutaneous insulin therapy and the ongoing complications that patients with Wolcott-Rallison syndrome are subject to.

## 1. Introduction

Wolcott-Rallison syndrome (WRS) is a genetic disease with permanent neonatal diabetes mellitus (NDM) in all affected individuals. Most patients also present bone abnormalities, exocrine pancreatic dysfunction and/or failure, and acute liver failure (ALF). Though permanent NDM is the permanent feature of the disease, ALF is the primary life-threatening manifestation of the disease [1].

NDM can be permanent or transient, depending on the specific genetic mutation. Transient forms typically resolve as the child grows, allowing for withdrawal of treatment, whereas permanent NDM requires lifelong administration of insulin of oral antidiabetic agents [2,3].

A particularly interesting form of transient NDM is caused by an imprinting imbalance concerning the genes at chromosome 6q24, which is the most common cause of transient NDM [2]. The syndrome is most likely due to dysfunction of the ZFP57 gene. This gene encodes a transcription factor involved in epigenetic regulation through the maintenance of appropriate levels of methylation on target genes [4]. Interestingly, there are case reports of relapses of diabetes mellitus in patients with 6q24-related NDM [5,6]. This suggests that environmental or other factors affecting epigenetic regulation play a role in the development of NDM, at least in part. Gestational diabetes mellitus has numerous environmental and epigenetic factors that predicts outcomes for both the mother and the fetus/infant [7,8] and more recent evidence has proposed that the hut microbiota influences the epigenetic control of genes relevant to NDM [9].

Other causes of transient NDM are mutations of genes that encode subunits of the ATP-sensitive potassium channel (either the KCNJ11 or ABCC8 genes), though some mutation can also cause permanent NDM [2]. This particular form of NDM typically responds well to treatment with oral sulphonylurea medications [10].

WRS is a very rare disease, with approximately 100 cases reported worldwide up until now. The largest cohorts have been described in the Middle East [11]. However, WRS is the most common form of permanent NDM [9].

WRS is caused by variants in the gene that encodes the eukaryotic translation initiation factor 2a kinase 3 (EIF2AK3) [1]. The protein is attached to the endoplasmic reticulum (ER) and plays an essential role in normal protein traffic through the ER, particularly during the translation phase of protein synthesis. Thus, when viewed from the standpoint of a disease that affects a protein associated with the normal production of other proteins in the ER, it is easy to understand that the core pathophysiological features of WRS are represented by dysfunctions of tissues that produce large quantities of proteins with various functions outside the individual cell. Permanent NDM is caused by gradual beta-cell death. Both endocrine and exocrine dysfunction lead to failure to thrive. Liver dysfunction predisposes patients with WRS to severe episodes of ALF. This report details the clinical characteristics, diagnosis, and management, and also highlights this rare diagnosis in a patient with non-consanguineous parents, unlike most cases reported thus far.

NDM poses serious challenges regarding management. Often, in the setting of diabetic ketoacidosis, intravenous insulin infusions are required to reestablish metabolic control. After the acute period, some patients, such as those with KCNJ11 or ABCC8 mutations, or those with 6q24-related NDM can respond well to sulphonylurea drugs (2). Of this class of drugs, glibenclamide has been widely used for NDM, and there is substantial evidence that it even improves neurologic outcomes in children with NDM [10,11,12].

Children with other forms of NDM, such as the case presented in this report, will require life-long subcutaneous insulin, coupled with quality-of-life measures, such as insulin pumps and continuous glucose monitoring sensors [13,14].

## 2. Case Report: History, Acute Presentation, and Acute Management of the Patient

The case report will focus on our male patient, who had been born at 39 weeks of gestation, via vaginal delivery, with an Apgar score of 10 points, a birth weight of 3200 g (38th percentile) and a length of 53 cm (95th percentile), in June 2021. The pregnancy had been uneventful, except that the mother presented mild COVID-19 during the fourth month. In the maternity ward, as per current local practices, the patient received the BCG and anti-hepatitis B vaccines with no adverse effects.

The infant had no apparent health issues for the first two months of life, although the parents reported that the baby had been particularly fussy and irritable. Despite these unremarkable issues, the patient thrived with good weight gain and was fed with commercially available milk formulas. Several physicians consulted the child due to the parent’s concern regarding his perceived irritability, but no clinical signs suggesting disease were identified besides the constant fussiness. No blood work was attempted during these visits since the child appeared well on clinical consultation.

At 9 weeks of age, the patients presented fever (up to 39 °C), several loose stools, poor appetite, and incessant crying. The mother described a brief loss of responsiveness and hypotonia in the infant, prompting an immediate presentation to the local emergency department. Upon initial consultation, the patient weighed 4500 g (5th percentile for age). He had a blood glucose level of 930 mg/dL and a venous blood pH of 7.15 (Table 1). Renal and liver function tests were normal, and C-reactive protein was also normal.

Two boluses of normal saline were administered, and an intravenous (IV) drip with normal saline was initiated. The patient was transferred to our tertiary care pediatric hospital and was admitted to the intensive care unit within 4 h of the initial presentation at his local hospital.

Given the extremely high blood glucose levels, acidosis and ketonemia, a working diagnosis of severe diabetic ketoacidosis (DKA) was established, and fluid therapy and fast-acting intravenous insulin were initiated as per the International Society for Pediatric and Adolescent Diabetes (ISPAD) guidelines [12]. The patient had a central venous catheter inserted in his left femoral vein, used initially for fluid resuscitation and later to deliver maintenance fluids. Short-acting insulin analogs were delivered via a separate peripheral venous catheter (0.05 units/kg/h, given the patient’s weight of 4.5 kg).

Subsequent IV fluids were a mix of normal saline and dextrose solutions, following the standard ISPAD guidelines for DKA. Gradually, the patient started accepting oral feeds, but due to the initial concern of a possible underlying metabolic disease, these feeds were limited to oral solutions of water and dextrose.

The patient gradually improved over the next 48 h, with blood glucose levels dropping and stabilizing between 200–300 mg/dL and blood pH values returning to normal. The patient was alert, eating orally, and was transferred to the pediatric ward for further care and additional diagnostic work-up. By this stage, the patient had been placed on commercially available infant milk formula for organic aciduria (see further sections below for details) and was still receiving the short-acting insulin IV infusion described above.

## 3. Detailed Diagnostic Work-Up and Further Management

### 3.1. Approach to the Differential Diagnosis and Subsequent Work-Up

The systematic analysis of the differential diagnosis of our patient was very important for the correct medical management of his condition. Table 2 provides an overview of the diagnoses that were considered in the first days of the child’s presentation, the diagnostic work-up that went into exploring these different possibilities and the subsequent approach employed by the medical team once the results of our investigation came back.

For type 1 diabetes mellitus (T1DM), specific autoantibodies for pancreatic beta-cell autoimmunity were tested; specifically, GAD II, ICA, and IA2 autoantibodies. All three tests came back negative, arguing against a diagnosis of T1DM [13].

The spectrum of neonatal diabetes mellitus (NDM) consists of numerous variants that cause pancreatic dysfunction, usually associated with other abnormalities [3,4,5,6,7,8,9,10,11,12,13,14]. The patient presented with extreme hyperglycemia and ketoacidosis that responded well to insulin therapy. Thus, a commercially available targeted genetic panel (Invitae, San Francisco, CA, USA) involving 23 genes known to cause various forms of NDM was performed. The patient is a compound heterozygote for two different variants in both copies of the EIF2AK3 gene: EIF2AK3 c.496_497delinsTG (p.Gln166*) and EIF2AK3 c.495_498del (p.Asp165Glufs*35). Genetic testing of the parents revealed that the patient’s mother carries the EIF2AK3 c.496_497delinsTG (p.Gln166*), while the father is a carrier of the EIF2AK3 c.495_498del (p.Asp165Glufs*35) variant.

### 3.2. In-Patient Setting Management

The long-term management of the patient was focused on controlling his blood glucose levels with minimal patient discomfort and addressing exocrine pancreatic failure.

As the patient’s status improved and oral feeds were re-established, the patient began subcutaneous (SC) insulin therapy (Lantus^TM^, Sanofi), starting at low doses, initially 0.5 U/day. As the patient continued to present spikes of hyperglycemia, sometimes with values above 400 mg/dL (but no concurrent acidosis or ketonemia), especially after larger meal volumes, insulin therapy was supplemented with IV administration of rapid-acting insulin (aspart-insulin). At this stage, IV rapid-acting insulin was provided for limited durations, usually one to several hours, with at least hourly measurements of blood glucose levels. Rapid-acting insulin IV infusions were stopped once blood glucose dropped to 200 mg/dL or below.

The SC glargin-insulin dose was slowly increased over several days, reaching 4 U/day after the first week. After reviewing the patient’s blood glucose levels, it was decided to split this dose into 3 U in the morning and 1 U in the evenings. As doses of SC glargin-insulin increased, the patient did not require any more IV supplementation of rapid-acting insulin because most blood glucose values across the day remained at or below 200 mg/dL.

To address the patient’s exocrine pancreatic insufficiency, oral pancreatic enzyme supplementation was added with no more than 10,000 units of lipase/day. The patient’s loose stools soon improved and normalized, and he presented satisfactory weight gain. Additional supplementation of fat-soluble vitamins was provided via a commercially available multivitamin oral solution.

A trial of oral glibenclamide was initiated. However, after several days of receiving an oral glibenclamide solution, our patient showed no improvement, and the treatment was abandoned. Furthermore, the finding of pancreatic hypoplasia did not fit the pattern of the mutations responsive to glibenclamide [15].

To provide a good balance between our need to closely monitor the patient’s blood glucose levels and the patient’s need for comfort, the patient was fitted with a continuous glucose monitoring (CGM) device attached to his thigh. These devices are readily available commercially, and they can stay attached with minimal discomfort for several days to a few weeks and provide real-time monitoring of blood glucose levels. Furthermore, some models can be paired with smartphone apps for ease of use, thus making it easy for parents to monitor their child’s condition. In our opinion, fitting the patient with such a device provided a substantial benefit because repeated finger-pricks were stopped, reducing the patient’s and the parent’s distress.

The patient was discharged home with a working diagnosis of monogenic diabetes (since the results of the genetic tests were still pending at the time), pancreatic exocrine insufficiency and pancreatic hypoplasia. At home, treatment consisted of offering the daily regular SC glargin-insulin injections in two divided doses (3 U in the morning and 1 U at night), oral pancreatic enzyme supplementation and multivitamin solution supplementation.

After receiving the results of the genetic tests, a formal diagnosis of WRS was formulated [16,17].

### 3.3. Long-Term Follow-Up and Development

Subsequent follow-up revealed a thriving patient, with normal neuro-developmental findings up to the age of 18 months. Table 3 provides an overview of the patient’s somatic development in the 18 months of follow-up and adjustments made to his insulin therapy as he grew. We eventually added fast-acting insulin to the patient’s daily regimen for improved glycemic control.

At 7 months of age, 5 months after the initial presentation, the patient was admitted to the hospital following symptoms of a respiratory tract infection, poor appetite, and emesis. Emergency laboratory work-up revealed markedly elevated liver functions tests. Aspartate aminotransferase (AST) levels were 36 times above the upper limit of normal (ULN), and alanine aminotransferase (ALT) values were 53 times above ULN. Additional laboratory work-up provided no evidence of any sign of ALF. Supportive care was promptly initiated, and the symptoms and elevated liver function tests (LFTs) subsided within days.

At 11 months, the patient presented with an episode of fever without a focus and elevated LFTs (AST 18 × ULN and ALT 22 × ULN), as well as elevated total and direct bilirubin. Liver ultrasonography revealed a centrilobular pattern (also known as the “starry sky” appearance). Clinically, the patient remained stable with no outward signs of symptoms of ALF, though the LFTs continued to deteriorate for a few days. The maximum values reached were AST 44 × ULN and ALT 76 × ULN. Continuous IV rehydration was provided with normal saline, 10% dextrose, and intravenous acetyl-cysteine. The patient returned to baseline parameters within a week.

At 18 months, the patient received lispro- and glargin-insulin analogs, continued oral supplementation with fat-soluble vitamins and pancreatic enzyme replacement therapy (up to 2000 units of lipase/dose, no more than 5 doses/day), with satisfactory growth and normal neurological development.

## 4. Discussion

This patient presented initially with extremely severe ketoacidosis, and he ultimately required concerted efforts from multiple specialists to reach the diagnosis. Ongoing long-term follow-up presents significant challenges.

The initial presentation was an unclear constellation of signs and symptoms, the ongoing fussiness reported by the parents, the fever and abnormally loose stools, and the brief episode of hypotonia and loss of responsiveness. Pediatric health care providers commonly encounter these signs and symptoms, yet they are also relatively unspecific, entailing an extensive differential diagnosis. However, given the initial lab work showing extreme hyperglycemia, acidosis, elevated levels of ketones, and normal C-reactive protein, the differential diagnosis diverged on two different paths during the first days of patient management.

First, the clinicians considered the possibility of a disorder of intermediary metabolism, given the very young age, reported constant fussiness, a sepsis-like appearance during the first hours after the presentation and profound metabolic acidosis. On the other hand, hyperglycemia is not a typical presentation of many metabolic diseases, and repeated measurements of blood ammonia levels are normal.

Second, given a patient hada severe DKA, one must consider the possibility of T1DM. The patient presented with the classical blood work-up of a patient with profound DKA (acidosis, ketones present in a urine sample and elevated blood ketone levels, hyperglycemia) and improved rapidly after starting treatment with a classical regimen for DKA. Furthermore, HbA1c was 9.4% (normal values under 6.4%), indicating long-standing hyperglycemia and nominally a criterion for a positive diagnosis of T1DM [14]. On the other hand, T1DM is extremely unlikely at the patient’s age of 9 weeks.

The possibility of monogenic diabetes should be considered in any infant with consistent hyperglycemia, especially if the initial presentation is severe. An excellent description of NDM is provided by the ISPAD publications [14]. It is important to remember that many cases are represented by variants that may be amenable to treatment with glibenclamide or other oral sulphonylurea drugs. This provides a fast, comparatively easy and non-distressing way of treating patients while also functioning as a rudimentary diagnostic test. Once responsiveness to oral sulphonylurea drugs is established, or lack thereof, a large proportion of the mutations involved in NDM can be ruled out of the differential diagnosis.

A large proportion of NDM is due to variants of the KCNJ11 gene, which encodes a potassium channel essential for the normal regulation of insulin secretion. Though less frequent than the KCNJ11 variant, other types of variants are also responsive to sulphonylurea drugs [15]. Glibenclamide provides excellent control of blood sugar levels in most patients presenting with KCNJ11 mutations [16]. Additionally, it can be easily administered orally and presents no discomfort for the patient, unlike SC insulin injections. Putative complications could be more readily prevented and/or treated in the in-patient setting. Thus, it is a valid option for empirical treatment, even before the results of genetic testing confirm any specific mutation. As the result of the genetic testing was still pending, an additional therapeutic intervention of an oral sulphonylurea treatment was tried. Our patient received an oral glibenclamide solution, but there was no improvement in controlling the high glucose levels.

While the acute presentation of our patient was dominated by the DKA and the need to apply standard DKA rehydration and IV insulin protocols, the long-term natural history of WRS is dominated by the potentially life-threatening episodes of ALF. The case series reported thus far confirms that most patients experience liver dysfunctions, with ALF as the leading cause of mortality in patients with WRS [11]. Our patient experienced two episodes of elevated LFTs, both during episodes of febrile illness, but without ALF.

At 7 months, the patient had had the second dose of a hexavalent vaccine a day before symptom onset and was then admitted for a first episode of non-specific febrile illness and elevated LFTs. While a definitive causal link cannot be established in this situation, it does illustrate the potential perils accompanying a WRS diagnosis. While diabetes can be effectively managed with SC insulin therapy, life-threatening episodes of ALF occur in most patients [11,12,13,14,15,16,17,18]. Indeed, one case report in the medical literature of one patient presented with recurrent episodes of ALF after routine childhood vaccinations [18]. Careful consideration of the risks and benefits regarding further vaccination or exposure to potential common pathogens should be considered.

According to published data, patients with NDM respond well to long-acting insulin analogs (glargin-insulin). This insulin analog provides stable action across the day and minimizes the risk of hypoglycemia [3,4,5,6,7,8,9,10,11,12,13,14,15]. However, it is essential to note that the use of the glargine-insulin analog, in this case, was strictly off-label. It was provided after the healthcare providers reviewed similar published case reports, and the family was given thorough informed consent regarding using this product.

Indeed, on several occasions, due to intensive blood sugar level monitoring, unexplained episodes of relative hypoglycemia were discovered, with values of 50 or 60 mg/dL or rapidly dropping below 100 mg/dL, despite recent meals. While no formal testing of this occurrence happened, these lower values seemed clustered during the night when the patient did not feed as often or around the early morning hours. Patients with WRS need very frequent and accurate monitoring of glycemia: abnormal liver function leads to impaired gluconeogenesis [19,20]. Thus, they can have problems maintaining sufficient circulating glucose levels, explaining our patient’s recurrent hypoglycemia episodes, especially during the night (when meals were spaced further apart). However, no clinically relevant symptoms were present in our patient. A large series of patients from a German-Austrian database was recently published [20]. A comparison of our case report to the 11 patients reported by Welters et al. is presented in Table 4.

Patients with WRS should be screened and treated for exocrine pancreatic insufficiency. Our patient demonstrated loose stools and relatively poor weight gain before diagnosis. Simple oral administration of pancreatic enzyme supplements at the end of larger meals ultimately resolved these symptoms in our patient and provided substantial weight and length gain.

WRS, due to the mutation of the EIF2AK3 gene, was the most frequently identified causes of permanent NDM in families with consanguineous marriages [21]. Our patient is the product of a non-consanguineous marriage and is a compound heterozygote for mutations of EIF2AK3. Once heterozygosity was established in our patient, testing the parents provided the necessary evidence to establish this diagnosis.

One limitation is that the results of genetic testing took time to obtain, during which time the patient carried a diagnosis of NDM, pending genetic results. Furthermore, once the compound heterozygous state of the patient became known, testing the parents also took time to confirm the diagnosis with certainty.

## 5. Conclusions

Neonatal diabetes mellitus is a consequence of many possible variants. The possibility of NDM should be seriously considered for any newborn or infant presenting with hyperglycemia. Additional features in each patient, such as a response to oral glibenclamide, liver dysfunction, or morphological abnormalities of other organs, can provide clues to specific conditions involving particular genes. Genetic testing established the diagnosis for our patient and provided valuable insights into long-term follow-up needs. Additionally, testing the parents confirmed their status as carriers of the two variants that comprise our patient’s compound-heterozygous status. Thus, our patient is the product of a non-consanguineous marriage, unlike most patients described in the medical literature. Future publications should focus on compiling information from individual case reports and efforts to include patients with WRS and NDM more broadly into databases, which would provide an additional benefit for understanding and treating these complex conditions.

## Figures and Tables

**Table 1:** Glycemia, blood pH and other parameters during the first hours after admission.

Parameter	1st h *	4th h	7th h	15th h
Glycemia (mg/dL)	930	621	519	491
Blood pH (venous)	7.05	6.88	6.93	7.14
Anion Gap (mmol/L)	-	28	31	34
Bicarbonate (mmol/L)	4.4	3.2	4.8	4.4
Base excess (mmol/L)	−26	−30	−27	−24
Ketones (mmol/L)	present in urine	3.6	-	-

* Parameters obtained during the first hour after the presentation in the local hospital.

**Table 2:** Differential diagnoses considered and therapeutic approaches.

Differential Diagnosis	Lab Test/Investigation	Results in Our Patient	Therapeutic Approach
Disorder of intermediary metabolism (DIM)	Plasma amino acid profile and plasma and urine organic acid	Normal	IV fluids (dextrose and normal saline solutions)
Type 1 diabetes mellitus (T1DM)	Pancreatic beta cell autoantibodies C-peptide levels	Negative for autoantibodies C peptide levels low and low-normal	Management of DKA as per ISPAD guidelines
Neonatal diabetes mellitus (NDM)	Genetic panel testing for NDM	Compound heterozygosity for the specific mutation	Initially: management of DKA as per ISPAD guidelines; A trial of oral glibenclamide—pending the results of genetic panel testing Glargine-insulin therapy detailed in the text
Persistent diarrhea	Abdominal ultrasound Stool sample testing for pancreatic elastase	Pancreatic hypoplasia Low elastase activity on repeated measurements	Oral pancreatic enzyme supplementation

**Table 3:** Somatic growth and insulin management during 18 months of follow-up.

Age (Months)	Weight, kg (Percentile)	Length, cm (Percentile)	HbA1c	Glargin-Insulin (UI/day)	Lispro-Insulin (UI/day)	Other Events (See Text for Details)
4 months	6.3 (p18)	63 (p34)	7.9%	5	-	-
6 months	8.12 (p58)	70.5 (p87)	8.1%	5	-	-
7 months	8.6 (p63)	72 (p90)	7.8%	5	-	URTI, hepatitis
9 months	9.2 (p62)	75 (p94)	7.3%	3.5	-	Nocturnal hypoglycemia
11 months	9.1 (p38)	78 (p93)	8%	5	-	Fever, hepatitis
13 months	9.4 (p33)	N/A	7.4%	5	-	-
14 months	9.5 (p29)	N/A	7.1%	4	-	Nocturnal hypoglycemia
16 months	9.15 (p10)	N/A	7.5%	3	3 (divided at 3 meals)	-
17 months	9.2 (p8)	78 (p11)	8.4%	4	6 (divided at 3 meals)	-
18 months	10 (p21	78 (p6)	8.3%	4	4.5–6 (divided at 3 meals)	-

HbA1c, glycated hemoglobin; URTI, upper respiratory tract infection; N/A, not available.

**Table 4:** Comparison of the current case report to the German-Austrian database published [20].

	At Onset			Latest-Follow-Up		
Current case report	Age	HbA1c	DKA	Age	IU/kg/day	HbA1c
	2 months old (0.16 years old)	9.4%	Yes	18 months	0.85–1	8.3%
German-Austrian cases (11 patients) [12]	0.28 yo (mean) 10 of 11 patients presented before 0.3 yo	8.1% (range 5.6–12.7%)	7 of 11 patients	range 1–15.5 yo 3 of the 11 patients deceased	0.82 (mean)	8.1% (mean)

IU—insulin units; yo—years-old.

## Data Availability

Not applicable.
